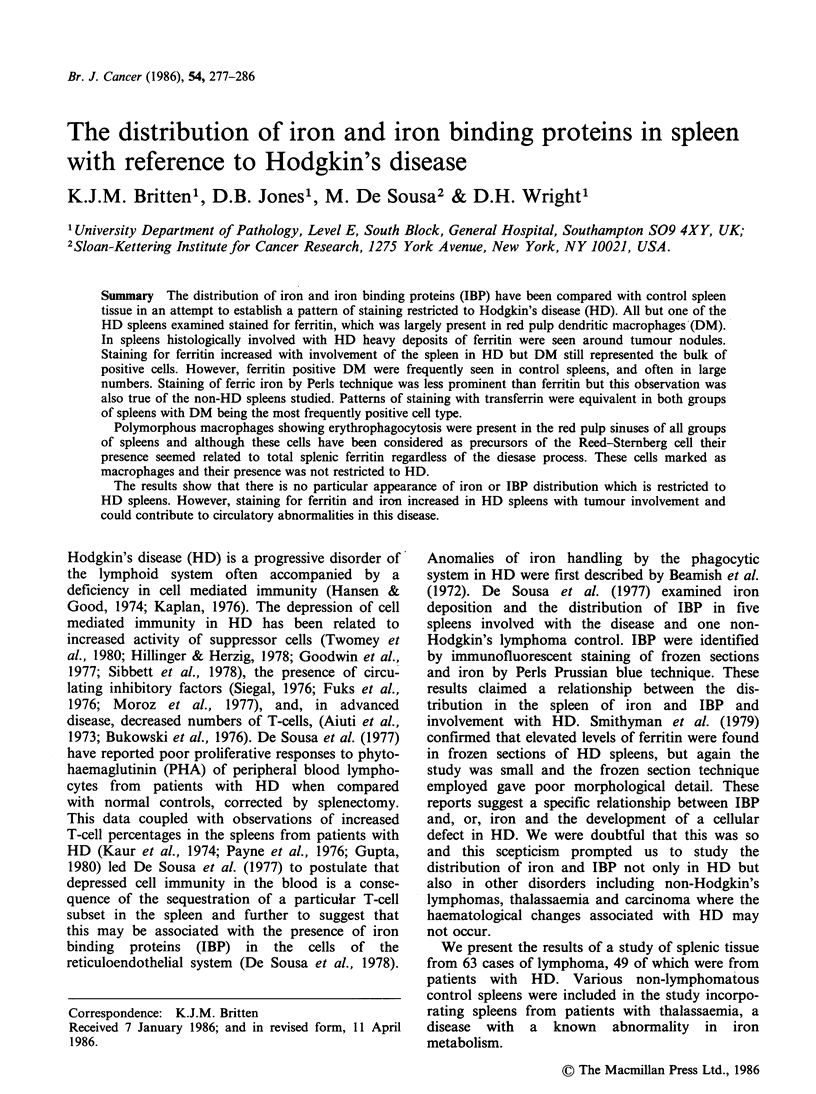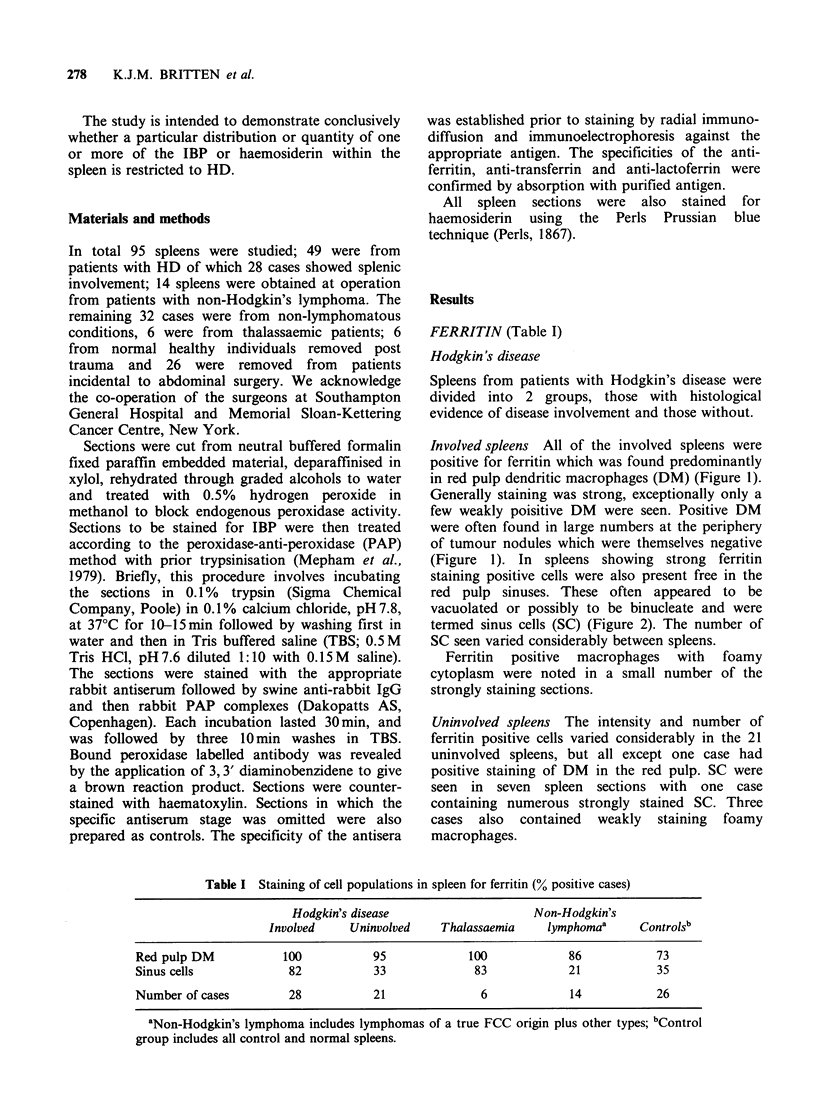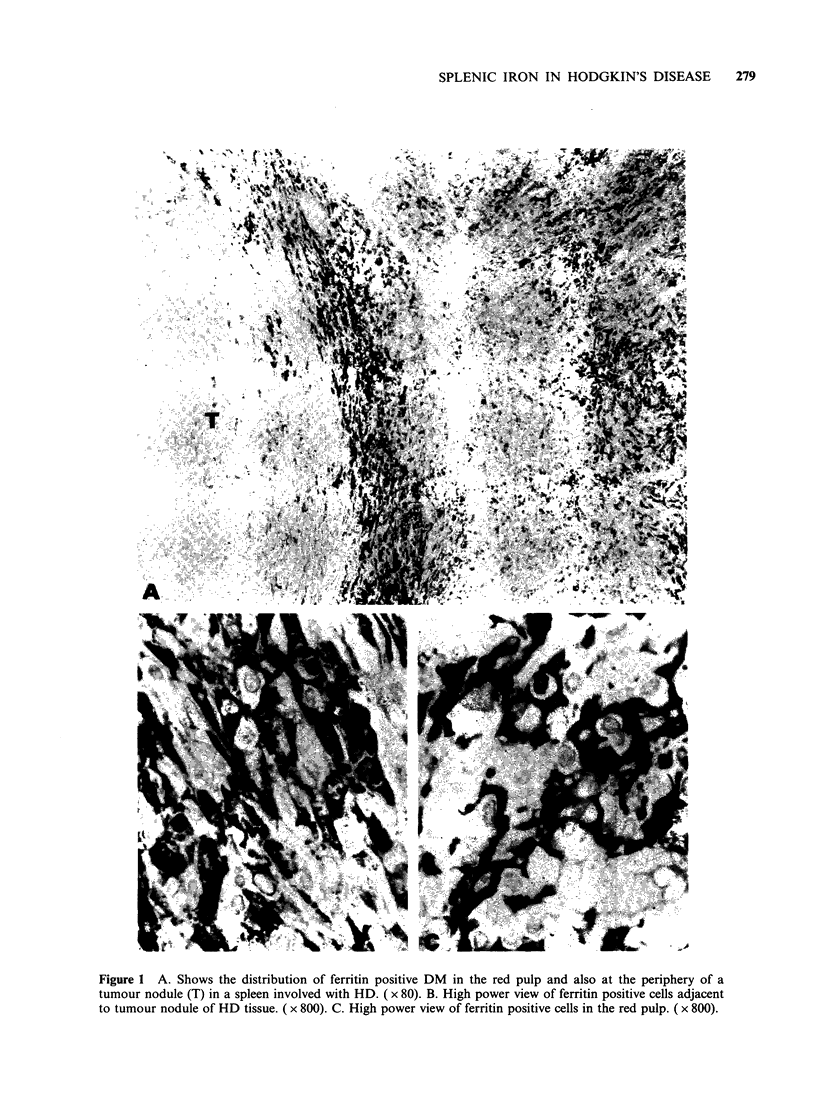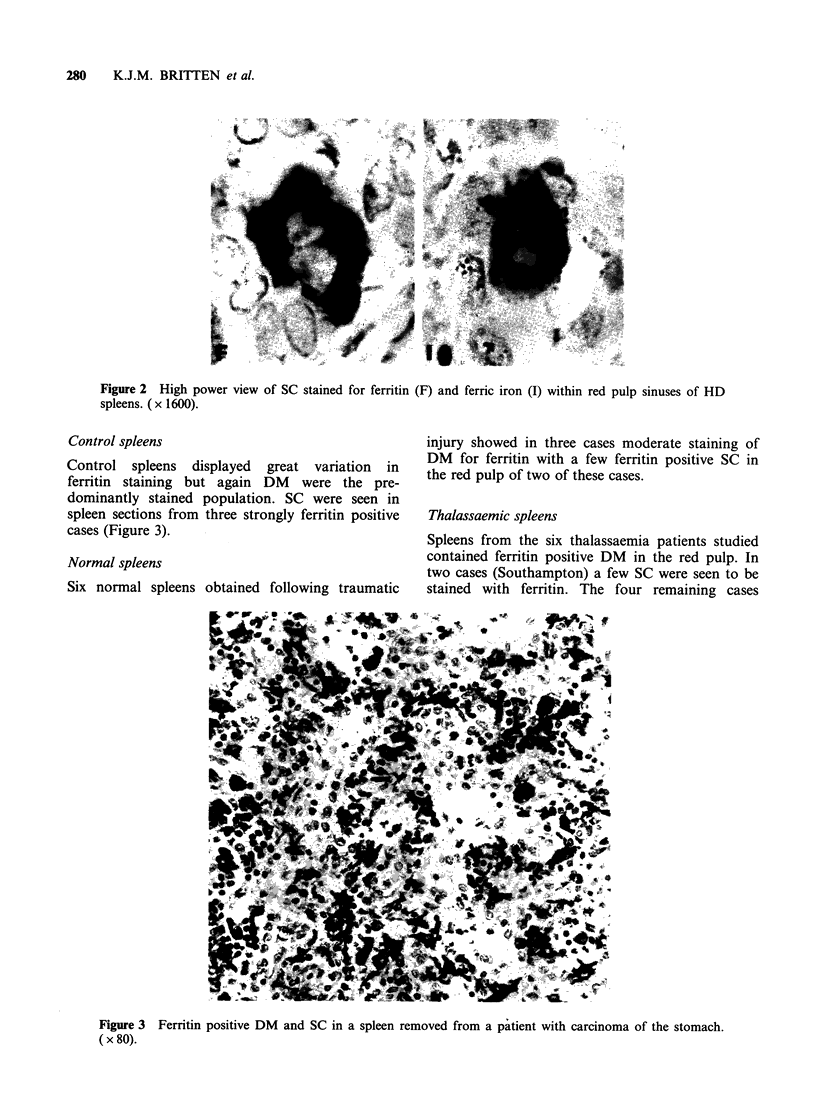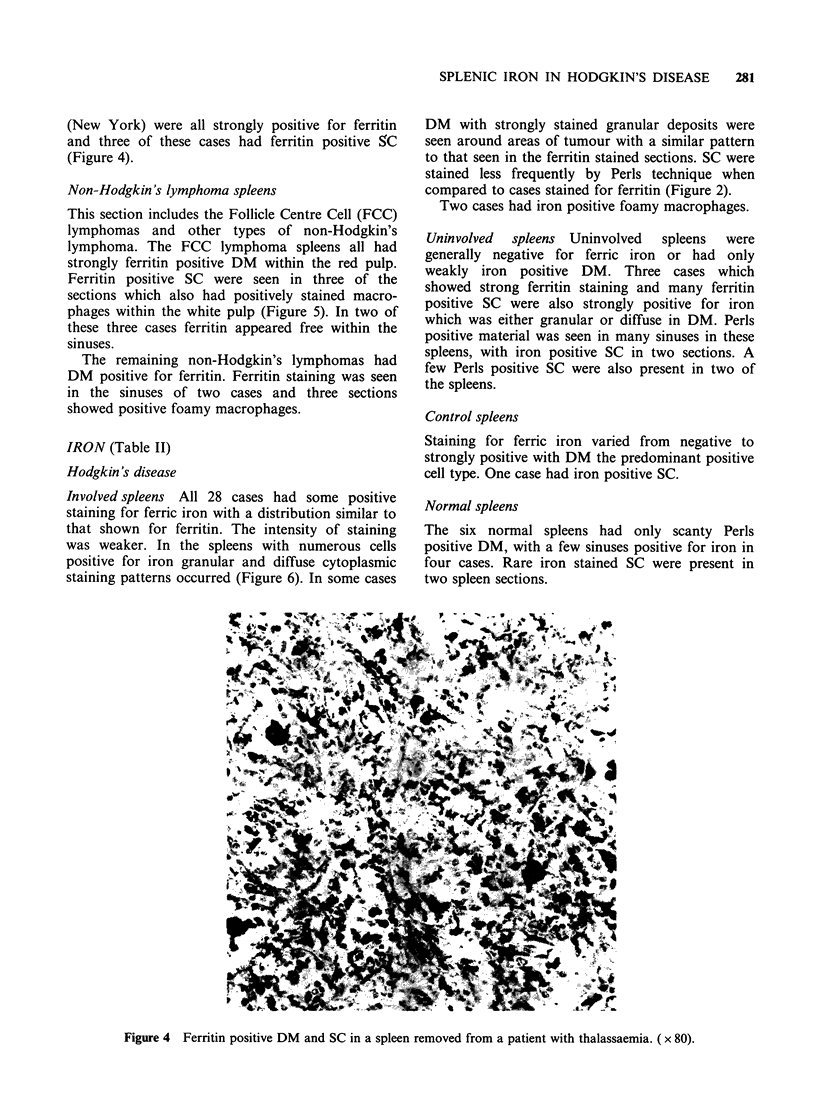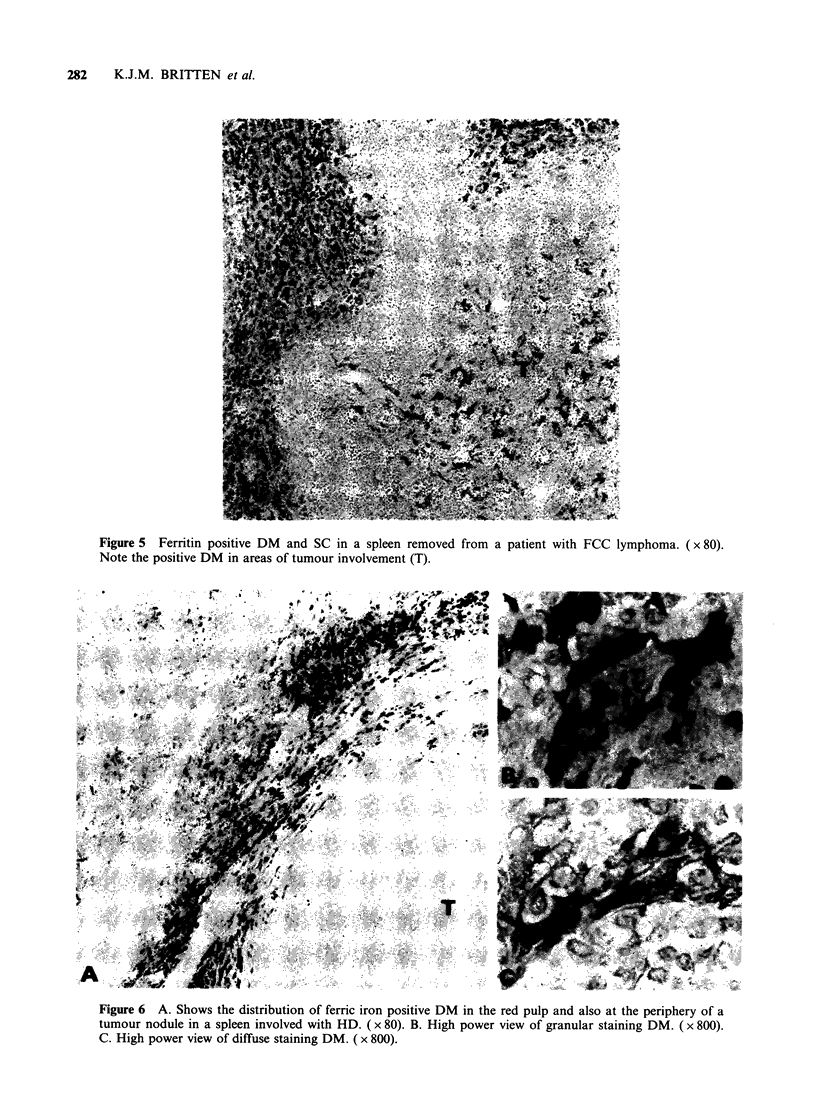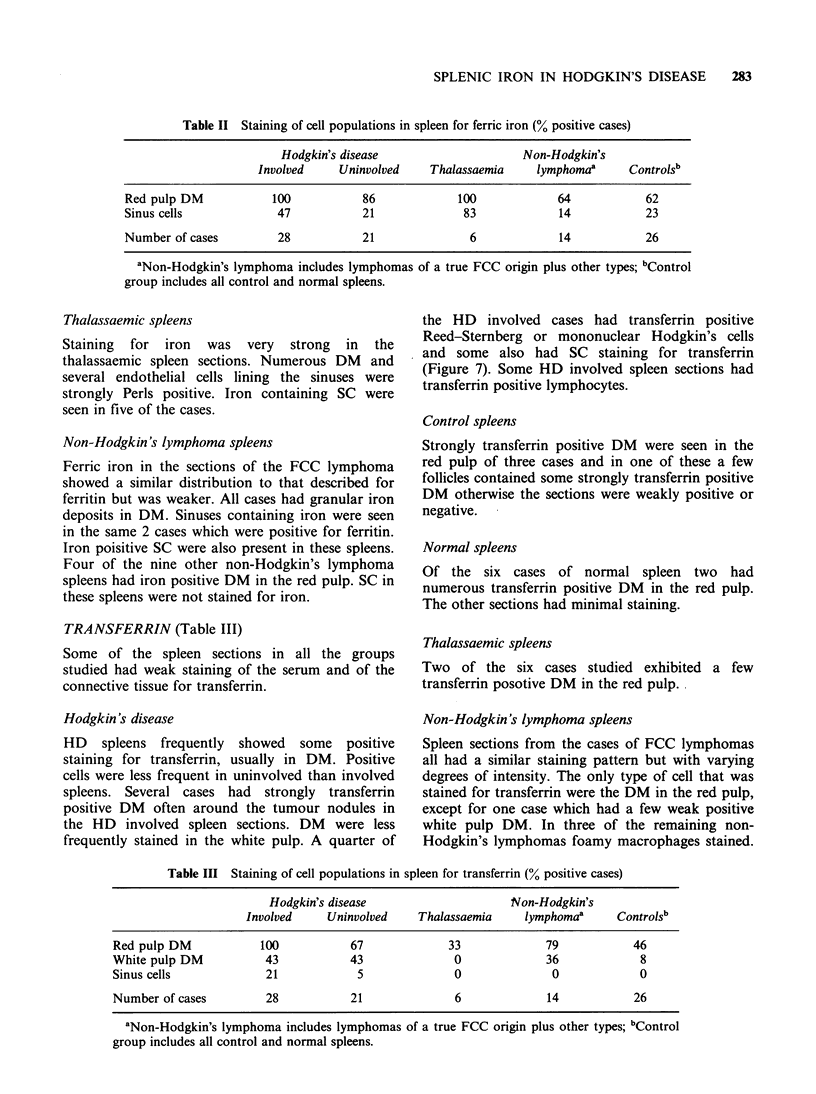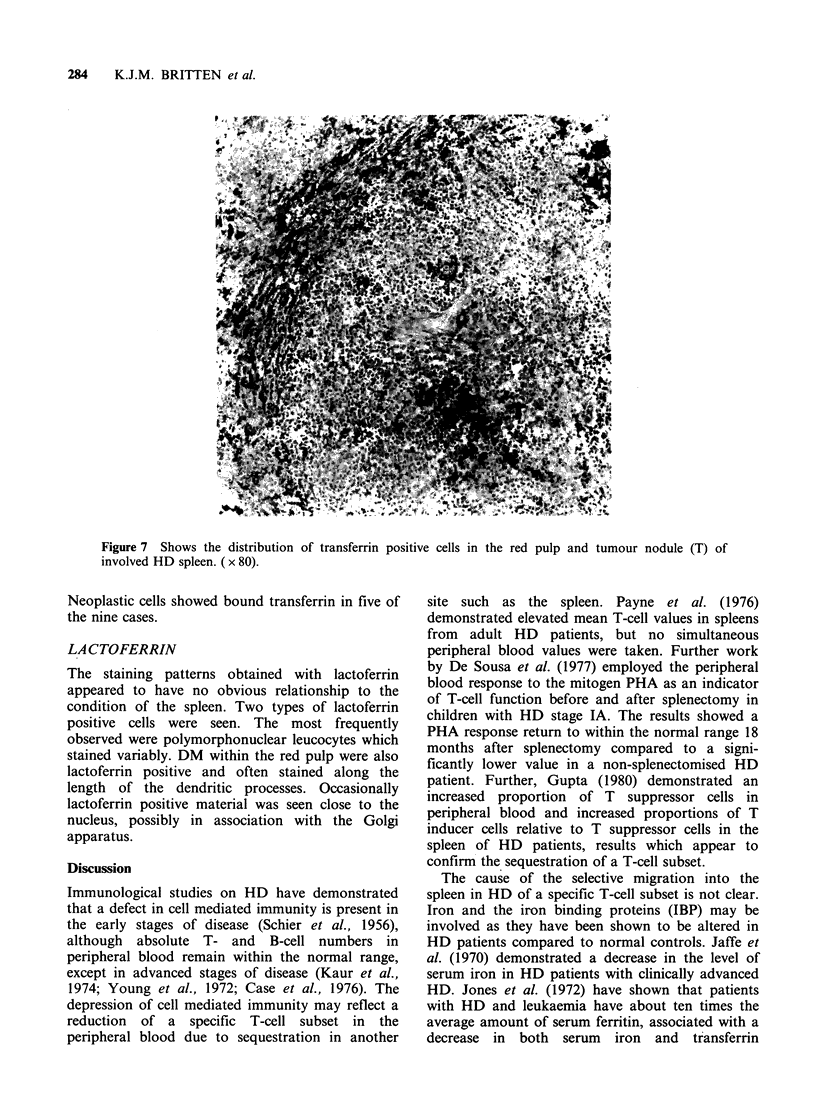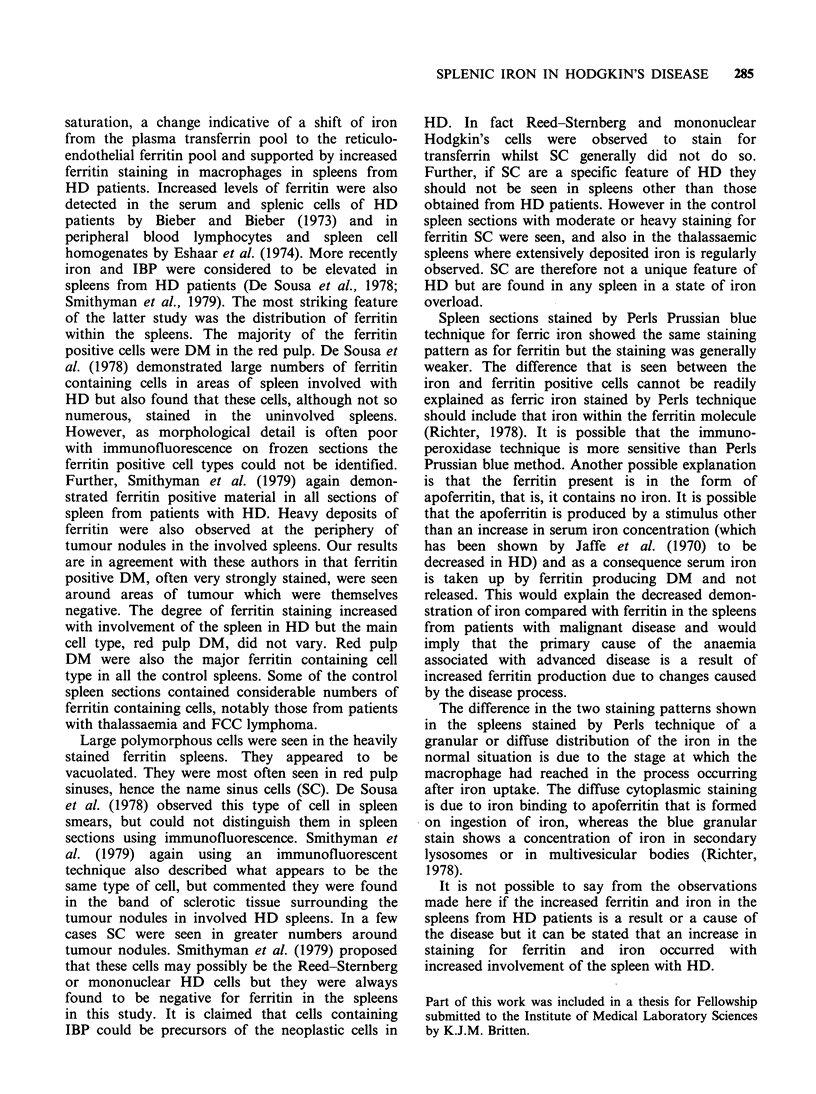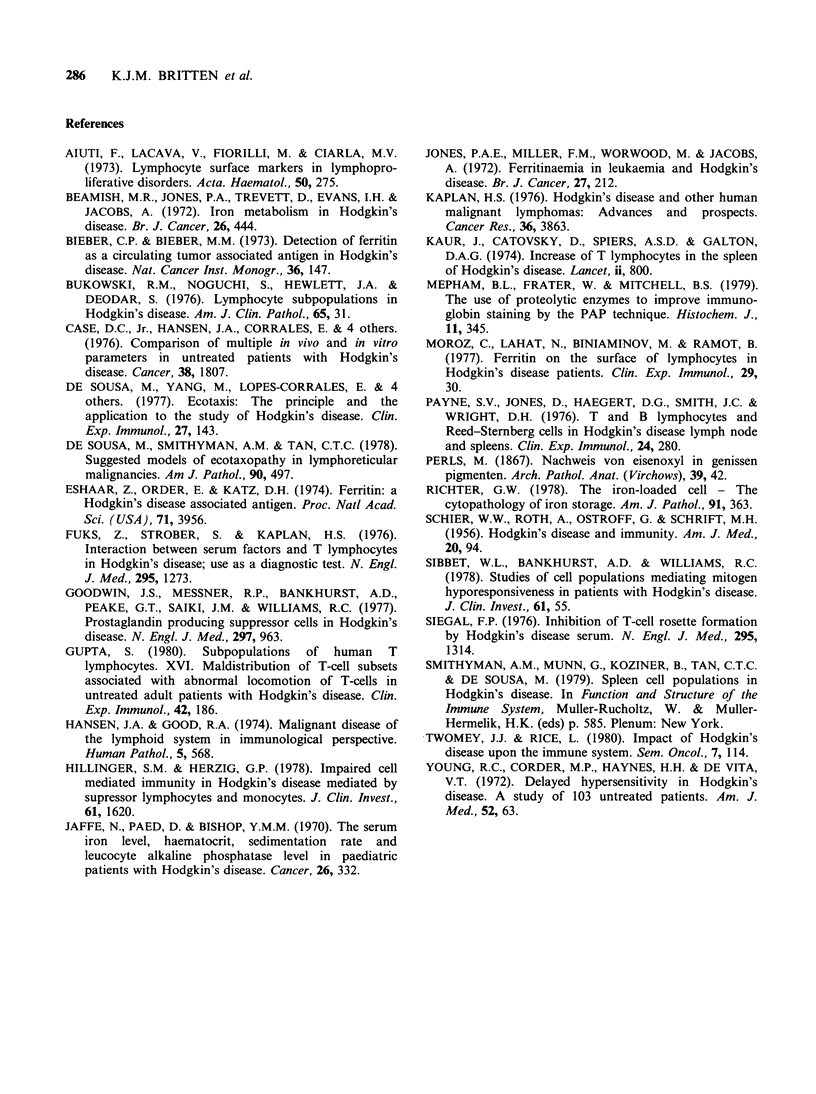# The distribution of iron and iron binding proteins in spleen with reference to Hodgkin's disease.

**DOI:** 10.1038/bjc.1986.174

**Published:** 1986-08

**Authors:** K. J. Britten, D. B. Jones, M. De Sousa, D. H. Wright

## Abstract

**Images:**


*Br. J. Cancer* (1986), **54**, 277–286

### The distribution of iron and iron binding proteins in spleen with reference to Hodgkin's disease

K.J.M. Britten[1], D.B. Jones[1], M. De Sousa[2] & D.H. Wright[1]

[1]*University Department of Pathology, Level E, South Block, General Hospital, Southampton SO9 4XY, UK;*
[2]*Sloan-Kettering Institute for Cancer Research, 1275 York Avenue, New York, NY 10021, USA.*

**Summary** The distribution of iron and iron binding proteins (IBP) have been compared with control spleen tissue in an attempt to establish a pattern of staining restricted to Hodgkin's disease (HD). All but one of the HD spleens examined stained for ferritin, which was largely present in red pulp dendritic macrophages (DM). In spleens histologically involved with HD heavy deposits of ferritin were seen around tumour nodules. Staining for ferritin increased with involvement of the spleen in HD but DM still represented the bulk of positive cells. However, ferritin positive DM were frequently seen in control spleens, and often in large numbers. Staining of ferric iron by Perls technique was less prominent than ferritin but this observation was also true of the non-HD spleens studied. Patterns of staining with transferrin were equivalent in both groups of spleens with DM being the most frequently positive cell type.

Polymorphous macrophages showing erythrophagocytosis were present in the red pulp sinuses of all groups of spleens and although these cells have been considered as precursors of the Reed–Sternberg cell their presence seemed related to total splenic ferritin regardless of the disease process. These cells marked as macrophages and their presence was not restricted to HD.

The results show that there is no particular appearance of iron or IBP distribution which is restricted to HD spleens. However, staining for ferritin and iron increased in HD spleens with tumour involvement and could contribute to circulatory abnormalities in this disease.

Hodgkin's disease (HD) is a progressive disorder of the lymphoid system often accompanied by a deficiency in cell mediated immunity (Hansen & Good, 1974; Kaplan, 1976). The depression of cell mediated immunity in HD has been related to increased activity of suppressor cells (Twomey *et al.*, 1980; Hillinger & Herzig, 1978; Goodwin *et al.*, 1977; Sibbett *et al.*, 1978), the presence of circulating inhibitory factors (Siegal, 1976; Fuks *et al.*, 1976; Moroz *et al.*, 1977), and, in advanced disease, decreased numbers of T-cells, (Aiuti *et al.*, 1973; Bukowski *et al.*, 1976). De Sousa *et al.* (1977) have reported poor proliferative responses to phytohaemaglutinin (PHA) of peripheral blood lymphocytes from patients with HD when compared with normal controls, corrected by splenectomy. This data coupled with observations of increased T-cell percentages in the spleens from patients with HD (Kaur *et al.*, 1974; Payne *et al.*, 1976; Gupta, 1980) led De Sousa *et al.* (1977) to postulate that depressed cell immunity in the blood is a consequence of the sequestration of a particular T-cell subset in the spleen and further to suggest that this may be associated with the presence of iron binding proteins (IBP) in the cells of the reticuloendothelial system (De Sousa *et al.*, 1978).

Correspondence: K.J.M. Britten
Received 7 January 1986; and in revised form, 11 April 1986.

Anomalies of iron handling by the phagocytic system in HD were first described by Beamish *et al.* (1972). De Sousa *et al.* (1977) examined iron deposition and the distribution of IBP in five spleens involved with the disease and one non-Hodgkin's lymphoma control. IBP were identified by immunofluorescent staining of frozen sections and iron by Perls Prussian blue technique. These results claimed a relationship between the distribution in the spleen of iron and IBP and involvement with HD. Smithyman *et al.* (1979) confirmed that elevated levels of ferritin were found in frozen sections of HD spleens, but again the study was small and the frozen section technique employed gave poor morphological detail. These reports suggest a specific relationship between IBP and, or, iron and the development of a cellular defect in HD. We were doubtful that this was so and this scepticism prompted us to study the distribution of iron and IBP not only in HD but also in other disorders including non-Hodgkin's lymphomas, thalassaemia and carcinoma where the haematological changes associated with HD may not occur.

We present the results of a study of splenic tissue from 63 cases of lymphoma, 49 of which were from patients with HD. Various non-lymphomatous control spleens were included in the study incorporating spleens from patients with thalassaemia, a disease with a known abnormality in iron metabolism.

© The Macmillan Press Ltd., 1986

The study is intended to demonstrate conclusively whether a particular distribution or quantity of one or more of the IBP or haemosiderin within the spleen is restricted to HD.

### Materials and methods

In total 95 spleens were studied; 49 were from patients with HD of which 28 cases showed splenic involvement; 14 spleens were obtained at operation from patients with non-Hodgkin's lymphoma. The remaining 32 cases were from non-lymphomatous conditions, 6 were from thalassaemic patients; 6 from normal healthy individuals removed post trauma and 26 were removed from patients incidental to abdominal surgery. We acknowledge the co-operation of the surgeons at Southampton General Hospital and Memorial Sloan-Kettering Cancer Centre, New York.

Sections were cut from neutral buffered formalin fixed paraffin embedded material, deparaffinised in xylol, rehydrated through graded alcohols to water and treated with 0.5% hydrogen peroxide in methanol to block endogenous peroxidase activity. Sections to be stained for IBP were then treated according to the peroxidase-anti-peroxidase (PAP) method with prior trypsinisation (Mepham *et al.*, 1979). Briefly, this procedure involves incubating the sections in 0.1% trypsin (Sigma Chemical Company, Poole) in 0.1% calcium chloride, pH 7.8, at 37°C for 10–15 min followed by washing first in water and then in Tris buffered saline (TBS; 0.5 M Tris HCl, pH 7.6 diluted 1:10 with 0.15 M saline). The sections were stained with the appropriate rabbit antiserum followed by swine anti-rabbit IgG and then rabbit PAP complexes (Dakopatts AS, Copenhagen). Each incubation lasted 30 min, and was followed by three 10 min washes in TBS. Bound peroxidase labelled antibody was revealed by the application of 3, 3′ diaminobenzidene to give a brown reaction product. Sections were counter-stained with haematoxylin. Sections in which the specific antiserum stage was omitted were also prepared as controls. The specificity of the antisera was established prior to staining by radial immuno-diffusion and immunoelectrophoresis against the appropriate antigen. The specificities of the anti-ferritin, anti-transferrin and anti-lactoferrin were confirmed by absorption with purified antigen.

All spleen sections were also stained for haemosiderin using the Perls Prussian blue technique (Perls, 1867).

### Results

*FERRITIN* (Table I)

*Hodgkin's disease*

Spleens from patients with Hodgkin's disease were divided into 2 groups, those with histological evidence of disease involvement and those without.

*Involved spleens* All of the involved spleens were positive for ferritin which was found predominantly in red pulp dendritic macrophages (DM) (Figure 1). Generally staining was strong, exceptionally only a few weakly poisitive DM were seen. Positive DM were often found in large numbers at the periphery of tumour nodules which were themselves negative (Figure 1). In spleens showing strong ferritin staining positive cells were also present free in the red pulp sinuses. These often appeared to be vacuolated or possibly to be binucleate and were termed sinus cells (SC) (Figure 2). The number of SC seen varied considerably between spleens.

Ferritin positive macrophages with foamy cytoplasm were noted in a small number of the strongly staining sections.

*Uninvolved spleens* The intensity and number of ferritin positive cells varied considerably in the 21 uninvolved spleens, but all except one case had positive staining of DM in the red pulp. SC were seen in seven spleen sections with one case containing numerous strongly stained SC. Three cases also contained weakly staining foamy macrophages.

**Table I** Staining of cell populations in spleen for ferritin (% positive cases)

| | *Hodgkin's disease* | | | *Non-Hodgkin's* | |
|---|---|---|---|---|---|
| | *Involved* | *Uninvolved* | *Thalassaemia* | *lymphoma*[a] | *Controls*[b] |
| Red pulp DM | 100 | 95 | 100 | 86 | 73 |
| Sinus cells | 82 | 33 | 83 | 21 | 35 |
| Number of cases | 28 | 21 | 6 | 14 | 26 |

[a]Non-Hodgkin's lymphoma includes lymphomas of a true FCC origin plus other types; [b]Control group includes all control and normal spleens.

**Figure 1** A. Shows the distribution of ferritin positive DM in the red pulp and also at the periphery of a tumour nodule (T) in a spleen involved with HD. (×80). B. High power view of ferritin positive cells adjacent to tumour nodule of HD tissue. (×800). C. High power view of ferritin positive cells in the red pulp. (×800).

**Figure 2** High power view of SC stained for ferritin (F) and ferric iron (I) within red pulp sinuses of HD spleens. ( × 1600).

*Control spleens*

Control spleens displayed great variation in ferritin staining but again DM were the predominantly stained population. SC were seen in spleen sections from three strongly ferritin positive cases (Figure 3).

*Normal spleens*

Six normal spleens obtained following traumatic injury showed in three cases moderate staining of DM for ferritin with a few ferritin positive SC in the red pulp of two of these cases.

*Thalassaemic spleens*

Spleens from the six thalassaemia patients studied contained ferritin positive DM in the red pulp. In two cases (Southampton) a few SC were seen to be stained with ferritin. The four remaining cases

**Figure 3** Ferritin positive DM and SC in a spleen removed from a patient with carcinoma of the stomach. ( × 80).

(New York) were all strongly positive for ferritin and three of these cases had ferritin positive SC (Figure 4).

*Non-Hodgkin's lymphoma spleens*

This section includes the Follicle Centre Cell (FCC) lymphomas and other types of non-Hodgkin's lymphoma. The FCC lymphoma spleens all had strongly ferritin positive DM within the red pulp. Ferritin positive SC were seen in three of the sections which also had positively stained macrophages within the white pulp (Figure 5). In two of these three cases ferritin appeared free within the sinuses.

The remaining non-Hodgkin's lymphomas had DM positive for ferritin. Ferritin staining was seen in the sinuses of two cases and three sections showed positive foamy macrophages.

*IRON* (Table II)

*Hodgkin's disease*

*Involved spleens* All 28 cases had some positive staining for ferric iron with a distribution similar to that shown for ferritin. The intensity of staining was weaker. In the spleens with numerous cells positive for iron granular and diffuse cytoplasmic staining patterns occurred (Figure 6). In some cases DM with strongly stained granular deposits were seen around areas of tumour with a similar pattern to that seen in the ferritin stained sections. SC were stained less frequently by Perls technique when compared to cases stained for ferritin (Figure 2).

Two cases had iron positive foamy macrophages.

*Uninvolved spleens* Uninvolved spleens were generally negative for ferric iron or had only weakly iron positive DM. Three cases which showed strong ferritin staining and many ferritin positive SC were also strongly positive for iron which was either granular or diffuse in DM. Perls positive material was seen in many sinuses in these spleens, with iron positive SC in two sections. A few Perls positive SC were also present in two of the spleens.

*Control spleens*

Staining for ferric iron varied from negative to strongly positive with DM the predominant positive cell type. One case had iron positive SC.

*Normal spleens*

The six normal spleens had only scanty Perls positive DM, with a few sinuses positive for iron in four cases. Rare iron stained SC were present in two spleen sections.

**Figure 4** Ferritin positive DM and SC in a spleen removed from a patient with thalassaemia. (×80).

**Figure 5** Ferritin positive DM and SC in a spleen removed from a patient with FCC lymphoma. (×80). Note the positive DM in areas of tumour involvement (T).

**Figure 6** A. Shows the distribution of ferric iron positive DM in the red pulp and also at the periphery of a tumour nodule in a spleen involved with HD. (×80). B. High power view of granular staining DM. (×800). C. High power view of diffuse staining DM. (×800).

**Table II** Staining of cell populations in spleen for ferric iron (% positive cases)

| | *Hodgkin's disease* Involved | *Uninvolved* | *Thalassaemia* | *Non-Hodgkin's lymphoma*[a] | *Controls*[b] |
|---|---|---|---|---|---|
| Red pulp DM | 100 | 86 | 100 | 64 | 62 |
| Sinus cells | 47 | 21 | 83 | 14 | 23 |
| Number of cases | 28 | 21 | 6 | 14 | 26 |

[a]Non-Hodgkin's lymphoma includes lymphomas of a true FCC origin plus other types; [b]Control group includes all control and normal spleens.

*Thalassaemic spleens*

Staining for iron was very strong in the thalassaemic spleen sections. Numerous DM and several endothelial cells lining the sinuses were strongly Perls positive. Iron containing SC were seen in five of the cases.

*Non-Hodgkin's lymphoma spleens*

Ferric iron in the sections of the FCC lymphoma showed a similar distribution to that described for ferritin but was weaker. All cases had granular iron deposits in DM. Sinuses containing iron were seen in the same 2 cases which were positive for ferritin. Iron poisitive SC were also present in these spleens. Four of the nine other non-Hodgkin's lymphoma spleens had iron positive DM in the red pulp. SC in these spleens were not stained for iron.

*TRANSFERRIN* (Table III)

Some of the spleen sections in all the groups studied had weak staining of the serum and of the connective tissue for transferrin.

*Hodgkin's disease*

HD spleens frequently showed some positive staining for transferrin, usually in DM. Positive cells were less frequent in uninvolved than involved spleens. Several cases had strongly transferrin positive DM often around the tumour nodules in the HD involved spleen sections. DM were less frequently stained in the white pulp. A quarter of the HD involved cases had transferrin positive Reed–Sternberg or mononuclear Hodgkin's cells and some also had SC staining for transferrin (Figure 7). Some HD involved spleen sections had transferrin positive lymphocytes.

*Control spleens*

Strongly transferrin positive DM were seen in the red pulp of three cases and in one of these a few follicles contained some strongly transferrin positive DM otherwise the sections were weakly positive or negative.

*Normal spleens*

Of the six cases of normal spleen two had numerous transferrin positive DM in the red pulp. The other sections had minimal staining.

*Thalassaemic spleens*

Two of the six cases studied exhibited a few transferrin posotive DM in the red pulp.

*Non-Hodgkin's lymphoma spleens*

Spleen sections from the cases of FCC lymphomas all had a similar staining pattern but with varying degrees of intensity. The only type of cell that was stained for transferrin were the DM in the red pulp, except for one case which had a few weak positive white pulp DM. In three of the remaining non-Hodgkin's lymphomas foamy macrophages stained.

**Table III** Staining of cell populations in spleen for transferrin (% positive cases)

| | *Hodgkin's disease* Involved | *Uninvolved* | *Thalassaemia* | *Non-Hodgkin's lymphoma*[a] | *Controls*[b] |
|---|---|---|---|---|---|
| Red pulp DM | 100 | 67 | 33 | 79 | 46 |
| White pulp DM | 43 | 43 | 0 | 36 | 8 |
| Sinus cells | 21 | 5 | 0 | 0 | 0 |
| Number of cases | 28 | 21 | 6 | 14 | 26 |

[a]Non-Hodgkin's lymphoma includes lymphomas of a true FCC origin plus other types; [b]Control group includes all control and normal spleens.

**Figure 7** Shows the distribution of transferrin positive cells in the red pulp and tumour nodule (T) of involved HD spleen. ( × 80).

Neoplastic cells showed bound transferrin in five of the nine cases.

*LACTOFERRIN*

The staining patterns obtained with lactoferrin appeared to have no obvious relationship to the condition of the spleen. Two types of lactoferrin positive cells were seen. The most frequently observed were polymorphonuclear leucocytes which stained variably. DM within the red pulp were also lactoferrin positive and often stained along the length of the dendritic processes. Occasionally lactoferrin positive material was seen close to the nucleus, possibly in association with the Golgi apparatus.

### Discussion

Immunological studies on HD have demonstrated that a defect in cell mediated immunity is present in the early stages of disease (Schier *et al.*, 1956), although absolute T- and B-cell numbers in peripheral blood remain within the normal range, except in advanced stages of disease (Kaur *et al.*, 1974; Young *et al.*, 1972; Case *et al.*, 1976). The depression of cell mediated immunity may reflect a reduction of a specific T-cell subset in the peripheral blood due to sequestration in another site such as the spleen. Payne *et al.* (1976) demonstrated elevated mean T-cell values in spleens from adult HD patients, but no simultaneous peripheral blood values were taken. Further work by De Sousa *et al.* (1977) employed the peripheral blood response to the mitogen PHA as an indicator of T-cell function before and after splenectomy in children with HD stage IA. The results showed a PHA response return to within the normal range 18 months after splenectomy compared to a significantly lower value in a non-splenectomised HD patient. Further, Gupta (1980) demonstrated an increased proportion of T suppressor cells in peripheral blood and increased proportions of T inducer cells relative to T suppressor cells in the spleen of HD patients, results which appear to confirm the sequestration of a T-cell subset.

The cause of the selective migration into the spleen in HD of a specific T-cell subset is not clear. Iron and the iron binding proteins (IBP) may be involved as they have been shown to be altered in HD patients compared to normal controls. Jaffe *et al.* (1970) demonstrated a decrease in the level of serum iron in HD patients with clinically advanced HD. Jones *et al.* (1972) have shown that patients with HD and leukaemia have about ten times the average amount of serum ferritin, associated with a decrease in both serum iron and transferrin saturation, a change indicative of a shift of iron from the plasma transferrin pool to the reticuloendothelial ferritin pool and supported by increased ferritin staining in macrophages in spleens from HD patients. Increased levels of ferritin were also detected in the serum and splenic cells of HD patients by Bieber and Bieber (1973) and in peripheral blood lymphocytes and spleen cell homogenates by Eshaar *et al.* (1974). More recently iron and IBP were considered to be elevated in spleens from HD patients (De Sousa *et al.*, 1978; Smithyman *et al.*, 1979). The most striking feature of the latter study was the distribution of ferritin within the spleens. The majority of the ferritin positive cells were DM in the red pulp. De Sousa *et al.* (1978) demonstrated large numbers of ferritin containing cells in areas of spleen involved with HD but also found that these cells, although not so numerous, stained in the uninvolved spleens. However, as morphological detail is often poor with immunofluorescence on frozen sections the ferritin positive cell types could not be identified. Further, Smithyman *et al.* (1979) again demonstrated ferritin positive material in all sections of spleen from patients with HD. Heavy deposits of ferritin were also observed at the periphery of tumour nodules in the involved spleens. Our results are in agreement with these authors in that ferritin positive DM, often very strongly stained, were seen around areas of tumour which were themselves negative. The degree of ferritin staining increased with involvement of the spleen in HD but the main cell type, red pulp DM, did not vary. Red pulp DM were also the major ferritin containing cell type in all the control spleens. Some of the control spleen sections contained considerable numbers of ferritin containing cells, notably those from patients with thalassaemia and FCC lymphoma.

Large polymorphous cells were seen in the heavily stained ferritin spleens. They appeared to be vacuolated. They were most often seen in red pulp sinuses, hence the name sinus cells (SC). De Sousa *et al.* (1978) observed this type of cell in spleen smears, but could not distinguish them in spleen sections using immunofluorescence. Smithyman *et al.* (1979) again using an immunofluorescent technique also described what appears to be the same type of cell, but commented they were found in the band of sclerotic tissue surrounding the tumour nodules in involved HD spleens. In a few cases SC were seen in greater numbers around tumour nodules. Smithyman *et al.* (1979) proposed that these cells may possibly be the Reed–Sternberg or mononuclear HD cells but they were always found to be negative for ferritin in the spleens in this study. It is claimed that cells containing IBP could be precursors of the neoplastic cells in HD. In fact Reed–Sternberg and mononuclear Hodgkin's cells were observed to stain for transferrin whilst SC generally did not do so. Further, if SC are a specific feature of HD they should not be seen in spleens other than those obtained from HD patients. However in the control spleen sections with moderate or heavy staining for ferritin SC were seen, and also in the thalassaemic spleens where extensively deposited iron is regularly observed. SC are therefore not a unique feature of HD but are found in any spleen in a state of iron overload.

Spleen sections stained by Perls Prussian blue technique for ferric iron showed the same staining pattern as for ferritin but the staining was generally weaker. The difference that is seen between the iron and ferritin positive cells cannot be readily explained as ferric iron stained by Perls technique should include that iron within the ferritin molecule (Richter, 1978). It is possible that the immunoperoxidase technique is more sensitive than Perls Prussian blue method. Another possible explanation is that the ferritin present is in the form of apoferritin, that is, it contains no iron. It is possible that the apoferritin is produced by a stimulus other than an increase in serum iron concentration (which has been shown by Jaffe *et al.* (1970) to be decreased in HD) and as a consequence serum iron is taken up by ferritin producing DM and not released. This would explain the decreased demonstration of iron compared with ferritin in the spleens from patients with malignant disease and would imply that the primary cause of the anaemia associated with advanced disease is a result of increased ferritin production due to changes caused by the disease process.

The difference in the two staining patterns shown in the spleens stained by Perls technique of a granular or diffuse distribution of the iron in the normal situation is due to the stage at which the macrophage had reached in the process occurring after iron uptake. The diffuse cytoplasmic staining is due to iron binding to apoferritin that is formed on ingestion of iron, whereas the blue granular stain shows a concentration of iron in secondary lysosomes or in multivesicular bodies (Richter, 1978).

It is not possible to say from the observations made here if the increased ferritin and iron in the spleens from HD patients is a result or a cause of the disease but it can be stated that an increase in staining for ferritin and iron occurred with increased involvement of the spleen with HD.

Part of this work was included in a thesis for Fellowship submitted to the Institute of Medical Laboratory Sciences by K.J.M. Britten.

### References

AIUTI, F., LACAVA, V., FIORILLI, M. & CIARLA, M.V. (1973). Lymphocyte surface markers in lymphoproliferative disorders. *Acta. Haematol.*, **50,** 275.

BEAMISH, M.R., JONES, P.A., TREVETT, D., EVANS, I.H. & JACOBS, A. (1972). Iron metabolism in Hodgkin's disease. *Br. J. Cancer,* **26,** 444.

BIEBER, C.P. & BIEBER, M.M. (1973). Detection of ferritin as a circulating tumor associated antigen in Hodgkin's disease. *Nat. Cancer Inst. Monogr.*, **36,** 147.

BUKOWSKI, R.M., NOGUCHI, S., HEWLETT, J.A. & DEODAR, S. (1976). Lymphocyte subpopulations in Hodgkin's disease. *Am. J. Clin. Pathol.*, **65,** 31.

CASE, D.C., Jr., HANSEN, J.A., CORRALES, E. & 4 others. (1976). Comparison of multiple *in vivo* and *in vitro* parameters in untreated patients with Hodgkin's disease. *Cancer,* **38,** 1807.

DE SOUSA, M., YANG, M., LOPES-CORRALES, E. & 4 others. (1977). Ecotaxis: The principle and the application to the study of Hodgkin's disease. *Clin. Exp. Immunol.*, **27,** 143.

DE SOUSA, M., SMITHYMAN, A.M. & TAN, C.T.C. (1978). Suggested models of ecotaxopathy in lymphoreticular malignancies. *Am J. Pathol.*, **90,** 497.

ESHAAR, Z., ORDER, E. & KATZ, D.H. (1974). Ferritin: a Hodgkin's disease associated antigen. *Proc. Natl Acad. Sci. (USA),* **71,** 3956.

FUKS, Z., STROBER, S. & KAPLAN, H.S. (1976). Interaction between serum factors and T lymphocytes in Hodgkin's disease; use as a diagnostic test. *N. Engl. J. Med.*, **295,** 1273.

GOODWIN, J.S., MESSNER, R.P., BANKHURST, A.D., PEAKE, G.T., SAIKI, J.M. & WILLIAMS, R.C. (1977). Prostaglandin producing suppressor cells in Hodgkin's disease. *N. Engl. J. Med.*, **297,** 963.

GUPTA, S. (1980). Subpopulations of human T lymphocytes. XVI. Maldistribution of T-cell subsets associated with abnormal locomotion of T-cells in untreated adult patients with Hodgkin's disease. *Clin. Exp. Immunol.*, **42,** 186.

HANSEN, J.A. & GOOD, R.A. (1974). Malignant disease of the lymphoid system in immunological perspective. *Human Pathol.*, **5,** 568.

HILLINGER, S.M. & HERZIG, G.P. (1978). Impaired cell mediated immunity in Hodgkin's disease mediated by supressor lymphocytes and monocytes. *J. Clin. Invest.*, **61,** 1620.

JAFFE, N., PAED, D. & BISHOP, Y.M.M. (1970). The serum iron level, haematocrit, sedimentation rate and leucocyte alkaline phosphatase level in paediatric patients with Hodgkin's disease. *Cancer,* **26,** 332.

JONES, P.A.E., MILLER, F.M., WORWOOD, M. & JACOBS, A. (1972). Ferritinaemia in leukaemia and Hodgkin's disease. *Br. J. Cancer,* **27,** 212.

KAPLAN, H.S. (1976). Hodgkin's disease and other human malignant lymphomas: Advances and prospects. *Cancer Res.*, **36,** 3863.

KAUR, J., CATOVSKY, D., SPIERS, A.S.D. & GALTON, D.A.G. (1974). Increase of T lymphocytes in the spleen of Hodgkin's disease. *Lancet,* **ii,** 800.

MEPHAM, B.L., FRATER, W. & MITCHELL, B.S. (1979). The use of proteolytic enzymes to improve immunoglobin staining by the PAP technique. *Histochem. J.*, **11,** 345.

MOROZ, C., LAHAT, N., BINIAMINOV, M. & RAMOT, B. (1977). Ferritin on the surface of lymphocytes in Hodgkin's disease patients. *Clin. Exp. Immunol.*, **29,** 30.

PAYNE, S.V., JONES, D., HAEGERT, D.G., SMITH, J.C. & WRIGHT, D.H. (1976). T and B lymphocytes and Reed–Sternberg cells in Hodgkin's disease lymph node and spleens. *Clin. Exp. Immunol.*, **24,** 280.

PERLS, M. (1867). Nachweis von eisenoxyl in genissen pigmenten. *Arch. Pathol. Anat.* (*Virchows*), **39,** 42.

RICHTER, G.W. (1978). The iron-loaded cell – The cytopathology of iron storage. *Am. J. Pathol.*, **91,** 363.

SCHIER, W.W., ROTH, A., OSTROFF, G. & SCHRIFT, M.H. (1956). Hodgkin's disease and immunity. *Am. J. Med.*, **20,** 94.

SIBBET, W.L., BANKHURST, A.D. & WILLIAMS, R.C. (1978). Studies of cell populations mediating mitogen hyporesponsiveness in patients with Hodgkin's disease. *J. Clin. Invest.*, **61,** 55.

SIEGAL, F.P. (1976). Inhibition of T-cell rosette formation by Hodgkin's disease serum. *N. Engl. J. Med.*, **295,** 1314.

SMITHYMAN, A.M., MUNN, G., KOZINER, B., TAN, C.T.C. & DE SOUSA, M. (1979). Spleen cell populations in Hodgkin's disease. In *Function and Structure of the Immune System,* Muller-Rucholtz, W. & Muller-Hermelik, H.K. (eds) p. 585. Plenum: New York.

TWOMEY, J.J. & RICE, L. (1980). Impact of Hodgkin's disease upon the immune system. *Sem. Oncol.*, **7,** 114.

YOUNG, R.C., CORDER, M.P., HAYNES, H.H. & DE VITA, V.T. (1972). Delayed hypersensitivity in Hodgkin's disease. A study of 103 untreated patients. *Am. J. Med.*, **52,** 63.